# The Moderating Effect of Occupational Burn-Out on the Link of Career Competencies to Career Sustainability Amid the COVID-19 Pandemic: A Mixed-Method Study

**DOI:** 10.3389/fpsyg.2022.916669

**Published:** 2022-07-12

**Authors:** Wei Zhang, Tachia Chin, Jian-Ben Peng, Yi-Nan Shan, George Kwame Agbanyo

**Affiliations:** College of Business, Honghe University, Mengzi, China

**Keywords:** burnout, career competencies, career sustainability, COVID-19, mixed-method

## Abstract

Due to the COVID-19 pandemic, most employees face increasing career-related stress, particularly those who work in multinational corporations (MNCs), because the international travel constraints prevent them from going back to their families. Hence, it is imperative to investigate the critical impact of employees’ occupational burnout (OB) on career-related outcomes. In response, this research explores the moderating effect of OB on the relationships between career competencies (CCs) and career sustainability (CS). To achieve a more comprehensive understanding of relevant issues, we adopted a mixed-method research design that includes both qualitative and quantitative analyses. Findings indicate that all the three dimensions of CCs (i.e., reflective, communicative, and behavioral career competencies; RC, CC, and BC) were positively related to CS; moreover, OB negatively moderated the individual relationships of CC and BC with CS. The main contribution of this study is to enrich the existing career literature by addressing the critical moderating role of OB in the links of CC and BC individually with CS. Practical implications are also discussed in this study.

## Introduction

With the spread of the COVID-19 pandemic, distorting global supply chains, people’s lives are largely affected around the world, particularly those working in multinational corporations (MNCs) (Chin et al., 2021; Zhang et al., 2022). In such a difficult time, an increasing number of burnout cases have occurred in MNCs as employees may have a great risk of losing their jobs. Burnout is defined as an occupational phenomenon where workers are not able to handle job stress properly, and it is also identified as a mental disease (World Health Organization [WHO], 2019). Although quite a few studies showed the negative impact of occupational burnout (OB) on the sustainability of careers (Chin and Rowley, 2018; Barthauer et al., 2019; Chin et al., 2019a; Torrès et al., 2021), it has become more critical for MNC managers to deal with employees’ OB amid an unprecedented global public health crisis (GPHC), such as the COVID-19. In response, this research is aimed to explore the role of burnout in affecting career-related issues during the pandemic.

Previous studies have examined the strong associations between career competencies (CCs) and career sustainability (CS) (Akkermans and Tims, 2017; Chin et al., 2021). Pieces of evidence indicated that CCs are positively related to CS (Chin et al., 2021; Zhang et al., 2022). Referring to Akkermans et al. (2013), the framework of CCs is actually composed of three individual dimensions, i.e., reflective competency (RC), communicative competency (CC), and behavioral career competency (BC) (Akkermans et al., 2013). However, given many existing studies considered CCs as an agglomerate variable (Kuijpers et al., 2006; Parker et al., 2009), there are scant studies investigating the effects of individual dimensions of CCs on CS. As a result, we focus on answering the question: What role does OB play in affecting the relationships between three dimensions of CCs and CS?

Taking together the foregoing arguments, the main contributions of this study are threefold. First, we address that the role of OB in the CC-CS associations in a particular GPHC context was riddled with volatility and uncertainty. Second, given that the use of only one method to explain the behaviors of employees is limited (Plomp et al., 2016; Hanif and Lallie, 2021), our mix-method research design partly enriches our understanding of the use of mixed-methods research techniques in career-related domains. Tertiary, from a cross-cultural contest, we collected data from Chinese employees working in Vietnam MNCs, which enrich the literature by using a unique sample with a cross-cultural setting in a particular GPHC context.

## Hypotheses Development

### Career Competencies and Career Sustainability

As well documented in the literature, CCs refers to the varying parameters of knowledge, skills, and abilities necessary for CS relative to the career demands and the personnel involvement (Akkermans et al., 2013, Zhang et al., 2022). Although many existing studies regard CCs as an agglomerate variable (Blokker et al., 2019; Cheng et al., 2022), recent research points out that it is more appropriate to see CCs as a composite measure that includes several dimensions; it is thus of great importance to test the individual dimensions of CCs on employee outcomes as this can provide a more comprehensive understanding of relevant issues (Chin et al., 2021). Hitherto, the most widely adopted CC measure, is proposed by Akkermans et al. (2013), which is composed of three dimensions, namely, RC, CC, and BC. We have also used this scale in this current research.

Relative competency emphasizes the cognitive significance of an individual’s “knowing why” or “self-knowledge skills.” Given that RC can be identified as the source of self-reflection and motivation toward individuals’ careers, it is very likely that Chinese employees will modify their perceptions of their CS in Vietnam under high uncertainty and complexity elicited by the COVID-19 as international travel constraints have made Chinese workers stuck in Vietnam and prevented them from going back to their hometowns. As such, we hypothesize:

**H1a.** RC of Chinese employees in Vietnam is positively related to their CS.

Communicative competency highlights the importance of social networking and self-presentation for seeking career guidance from others (Chin et al., 2019a; Ren et al., 2020). However, the GPHC has forced workers to wear face masks and reduce social contact with others to lower the infection risk at the workplace; cross-cultural conflicts may thus occur more easily and frequently between Chinese and Vietnam workers. In this vein, the possession of CC has become particularly critical for individuals to seek feedback from and collaborate with colleagues; hence, we hypothesize:

**H1b.** CC of Chinese employees in Vietnam is positively related to their CS.

Behavioral career competency refers to one’s ability to proactively take action to shape their career. While the COVID-19 has incurred chaos in the global labor market, it has become more critical for individuals to identify career-related opportunities. As a result, the possession of BC may help Chinese workers to better adapt to changing job environment in Vietnam.

**H1c.** BC of Chinese employees in Vietnam is positively related to CS.

### The Moderating Effect of Occupational Burnout

**FIGURE 1:**
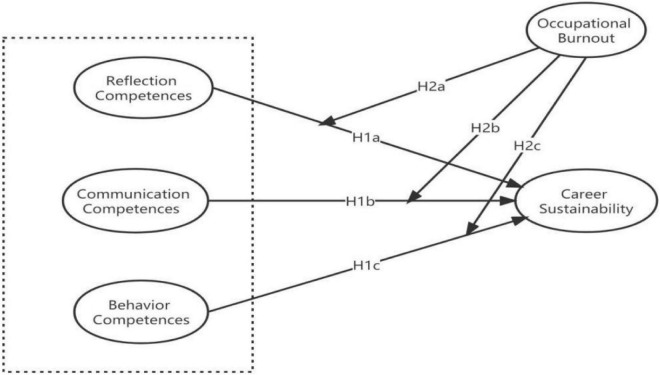
The research framework.

The advent of the COVID-19 pandemic has resulted in a mass job loss around the world. Workers not only have to deal with the threat of infection but also need to tackle financial problems caused by job losses (Baruch and Vardi, 2016). Previous studies have indicated the negative impact of OB on career (Chin and Liu, 2015; Barthauer et al., 2019; Chin et al., 2019b; Torrès et al., 2021). Following this logic, we advance to argue that OB may also deteriorate the assumed positive associations between CCs and CS. Accordingly, Figure 1 illustrates a comprehensive research framework of this study. In this spirit, we thus hypothesize:

**H2a.** The higher the level of OB, the weaker the relationship between RC and CS.
**H2b.** The higher the level of OB, the weaker the relationship between CC and CS.
**H2c.** The higher the level of OB, the weaker the relationship between BC and CS.

## Methodology

To achieve a deeper understanding of relevant issues, we adopted a mixed-method; two-phase research design that includes qualitative and quantitative approaches (Creswell, 2014). Qualitative methods allow researchers to explore the target phenomenon, discover essential constructs, and create a comprehensive grasp of the mechanism, while quantitative approaches are valuable for examining proposed hypotheses and hypothetical relationships (Venkatesh et al., 2013). Following this logic, we first conducted in-depth interviews with qualitative analysis to identify the hypothetical model, followed by a questionnaire survey with a larger sample to examine the theoretical relationships by quantitative analysis. In sum, employing such a mixed-method approach enabled us to collect diverse types of data to produce more convincing results.

## Study 1: Qualitative Research

### Sample and Data Collection

For the qualitative survey, we first conducted a case study in Vietnam Yuebei Nonferrous Metals Corporation, a China-invested MNC, owns two plants with over 2,000 manufacturing workers in Vietnam. Due to the impact of COVID-19, one of its factories in Bắc Ninh in Vietnam was closed down and over 600 people lost their job. At present, another factory in Thai Nguyen is also struggling to sustain the operation. Therefore, the workers who were survived from the layoffs became increasingly anxious about their job security. After interacting with their HR manager, we carried out in-depth interviews on five (5) job positions and their Chief Executive Officer (CEO); their demographic information, position, and tenure are illustrated in Table 1.

**TABLE 1:** Demographic information of participants.

No	Name	Education	Gender	Age	Tenure	Dept.	Position	Monthly wage (2021)
								(RMB and USD)
1	Participant 1	Master	M	61	35	CEO office	CEO	About, 520,000/81,700
2	Participant 2	Junior high	M	43	23	Production	Manager	32,000/5030.4
3	Participant 3	Master	F	36	14	Sales	Foreman	11,500/1807
4	Participant 4	College	F	31	6	Production	Foreman	10,500/1650.6
5	Participant 5	Secondary Vocational	M	28	2.5	Production	Worker	5,300/833.1
6	Participant 6	Junior high	F	23	2	Production	Worker	3,600/565.9

We spent 2 days doing online interviews with these five workers, which took between 45 and 85 min each, and we spent 90 min in the CEO’s office conducting face-to-face interviews. The following research questions guided the interview:

1. How do employees view the relationship between CS and the three individual dimensions of CCs?
  (a) How does RC [reflection on motivation (RCM) and qualities] impact CS?
  (b) How does CC [self-profiling (CCP) and networking (CCN)] impact CS?
  (c) How does BC [work exploration (BCW) and career control (BCC)] impact CS?
2. During the COVID-19 pandemic, what role does OB in influencing the relationship between CS and three individual dimensions of CCs?
  (a) How does OB interfere in the relationship between RC and CS?
  (b) How does OB interfere in the relationship between CC and CS?
  (c) How does OB interfere in the relationship between BC and CS?

The questions were made flexible for answering because they were based on the participants’ practical stories.

### Key Findings of Study 1

Our findings showed that all 6 participants agreed that each dimension of the CCs positively impacted in sustaining their careers (e.g., participant 2 stated: “After each work project is finished, I always write down my reflection on what can be done better. Moreover, I spend plenty of time learning new things related to my job spontaneously. With such a reflexive level of CCs, I have great confidence in my career development even in such difficult times.” participant 4 stated that “I am good at making friends with local people who help me expand the network of my customer base so that I can keep my job in the long run”; participant 5 stated: “Being stimulated by the goal of being a manager, I have sustained a consistently hardworking behavior with the belief that I will have a bright future in my career”). Moreover, according to the interviewers, their perceptions of OB had been strengthened during the pandemic, but it should be noted that their views on the role of OB affecting the relationship between CCs and CS are different. For instance, as participant 1 stated: “I felt that sociable employees are more likely to suffer from the OB in our company due to lockdown, and they had become more anxious about their job security.” In addition, the employees with clear career plans tended to be affected by the OB to develop a sustainable career. Contrarily, employees who have the good reflective ability, become more active in developing their careers even with the perception of OB. Similarly, participants 1, 2, and 6 also felt that OB has a negative influence on the relationship between CS, CC, and BC, respectively. However, they do not think OB will affect the relationship between RC and CS.

In conclusion, these findings suggested that our hypothesis development is rational and consistent with the respondents’ feedback. There is also a perception that OB affects the relationship between CCs’ individual dimensions and CS differently. In the following section, we conducted a quantitative survey to validate the hypotheses.

## Study 2: Quantitative Research

### Instrument Designing

In this study, we used the three dimensions of CCs (RC, CC, and BC) as independent variables. Moreover, we used CS as the dependent variable and OB as a moderator for the relationship between CCs and CS.

Independent variables of CCs were measured with the scale with 21-item developed by Akkermans et al. (2013). All the CC items were measured using the six-point Likert scale (1 = strongly disagree to 6 = strongly agree). RC, CC, and BC each were measured with 7 items. Sample items for RC were “I know what is important to me in my career” and “I am familiar with my shortcomings in my work”; sample items for CC were “I know how to ask for advice from people in my network” and “I can clearly show others what my strengths are in my work”; and sample items for BC were “I know how to seek for developments in my area of expertise” and “I can clearly show others what my strengths are in my work.”

The dependent variable CS was measured with a scale having 12 items developed by Chin et al. (2021). All the items of CS were measured using the six-point Likert scale (1 = strongly disagree to 6 = strongly agree). Sample items include “My career makes me feel like I have a bright future,” “My career gives me a lot of flexibility,” “My career provides me opportunities to update my skills,” and “My career builds my ability to absorb information and knowledge.”

The moderator OB was measured with the scale that have 6 items developed by Hakanen et al. (2006). Referring to the original research, the scale consisted of 16 items that comprised both positive and negative statements and was aimed at assessing burnout and engagement. We chose to utilize solely negatively phrased items to measure “pure” burnout since our research setting was under the GPHC. Additionally, the scale’s items were adopted and reduced to six. We used the six-point Likert scale to evaluate all the items of OB (1 = strongly disagree to 6 = strongly agree). Sample items included “Today, I thought less at work and did my job almost mechanically” and “Today during my work, I felt emotionally drained.”

To guarantee that all questions were understood, the survey was conducted in the Chinese language, with the questionnaire’s phrasing modified to the Chinese context. Moreover, to confirm the correctness of the translated items and to increase methodological rigor, we adopted the back-translation approach.

### Sample and Data Collection

Before formally starting the survey, we conducted in-depth interviews with 5 Chinese workers who have worked in Vietnam for over 5 years to ensure the appropriateness and clarity of our hypotheses and measures. Our assumptions were rational and consistent with reality, according to their feedback. With the assistance of the Business Association of China in Vietnam,^1^ we carried out a formal survey both online and offline from September to December 2021, the respondents to this study were Chinese employees working at MNCs in Vietnam. We used WJX,^2^ a professional online survey service in China, to conduct the online survey, we also asked one of the managers in Vietnam to distribute the questionnaires and scan the result for us. We explained the survey’s procedure confidentiality throughout the formal survey. No confidential information was requested, and all responses were voluntary. Finally, we acquired 204 usable data after eliminating 26 invalid samples.

Survey participants were Chinese workers from China-invested manufacturing MNCs in Vietnam. Of all respondents, about 82.3% were aged less than 40 years old; 63.23% were men and 36.77% were women; 45.76% were married, and 54.24% were unmarried. Additionally, about 73% of participants worked in Vietnam for 4 years or above. The respondents were from different manufacturing sectors: 33.43% from original equipment manufacturing, 23.81% from the textile industry, 19.86% from the electronic manufacturing, 13.16% from the auto parts manufacturing, and 9.74% from the machinery industry. Out of the 204, 8.08% had master’s degrees, 43.54% had bachelor’s degrees, and 43.38% had an associated degree or below. Moreover, nearly 52.4% of the MNCs where managers worked had less than 500 employees, and most of these MNCs are established in northern Vietnam (63.4%).

## Results

Statistical Package for the Social Sciences (SPSS) and SmartPLS 3.0 were used to analyze the data collected in our study. We utilized SPSS to analyze the demographic data and Smart PLS3.0 to examine the structural model’s reliability and convergent validity (Ringle et al., 2015).

### Common Method Variance

We employed the single-factor approach to test standard method variance [common method variance (CMV)]. Our research used self-reported data (Podsakoff et al., 2003). According to exploratory factor analysis, only 32.389% of the variance was explained by the first factor, which was lower than the threshold of 50%. We concluded that no significant standard method variance existed as the value was within the acceptable range.

### Reliability and Validity

Our study used Cronbach’s alpha and composite reliability (CR) to test the reliability. It should be noted that we have examined the reliability of each construct for the three independent variables, as shown in Table 2:

**TABLE 2:** Confirmatory factor analysis.

Construct	Items	Factor loading	α	rho_A	CR	AVE	VIF
Career Sustainability (CS)	CS1	0.733***	0.925	0.925	0.935	0.547	DV
	CS2	0.724***					
	CS3	0.766***					
	CS4	0.745***					
	CS5	0.727***					
	CS6	0.751***					
	CS7	0.743***					
	CS8	0.722***					
	CS9	0.768***					
	CS10	0.722***					
	CS11	0.715***					
	CS12	0.757***					
Reflection on motivation (RCM)	RC1	0.913***	0.902	0.905	0.939	0.837	1.000
	RC2	0.900***					
	RC3	0.930***					
Reflection on qualities (RCQ)	RC4	0.918***	0.927	0.929	0.948	0.820	1.000
	RC5	0.879***					
	RC6	0.895***					
	RC7	0.929***					
Networking (CCN)	CC1	0.900***	0.922	0.922	0.945	0.810	1.000
	CC2	0.886***					
	CC3	0.889***					
	CC4	0.925***					
Self-profiling (CCP)	CC5	0.918***	0.903	0.904	0.940	0.838	1.000
	CC6	0.910***					
	CC7	0.919***					
Work exploration (BCW)	BC1	0.896***	0.887	0.888	0.930	0.816	1.000
	BC2	0.900***					
	BC3	0.914***					
Career control (BCC)	BC4	0.923***	0.927	0.927	0.948	0.821	1.000
	BC5	0.877***					
	BC6	0.902***					
	BC7	0.922***					

Career sustainability (CS), reflection on motivation (RCM), reflection on qualities (RCQ), networking (CCN), self-profiling (CCP), work exploration (BCW), career control (BCC). ***Factoring loading > 0.7.

Reflective competency includes RCM and reflection on qualities (RCQ); CC includes CCN and CCP; BC includes BCW and BCC. Cronbach’s α coefficients for each dimension ranged from 0.887 to 0.927 (RCQ), which were higher than the acceptable value of 0.7 (Hair et al., 2019). Table 2 shows that CR for all constructs is well above the recommended value of 0.7 and ranges from 0.930 to 0.948 (RCQ, respectively). Moreover, rho A, a new indicator coefficient introduced in Smart PLS3.0, was tested to rectify the measured structure estimate. As shown in Table 2, rho A ranges from 0.888 to 0.929 (RCQ, respectively), which is higher than the required threshold of 0.7 (Dijkstra and Henseler, 2015; Hair et al., 2017).

We examined the convergent validity by verifying whether the factor loadings of all items were above 0.7 and the average variance extracted values were higher than 0.5 (Hair et al., 2017). As illustrated in Table 2, the factor loadings of all items range from 0.715 to 0.930 (RCM, respectively), and AVE ranges from 0.547 to 0.838 (CCP, respectively). Our measurement model thereby possessed an acceptable degree of convergent validity. Table 2 also shows that the variance inflation factor (VIF) values are lower than 5, indicating no collinearity issues. Discriminant validity was evaluated by using Heterotrait-Monotrait (HTMT) and ensuring that it is below 0.90 (Henseler et al., 2014). According to our study, the values of HTMT were ranged from 0.386 to 0.687, which indicated that our measurement model had acceptable discriminant validity.

Standardized root mean squared residual (SRMR) was employed in our study to check whether the research model is in a good fit. According to Henseler et al. (2014), the required value of SRMR is lower than 0.10. In our research, the SRMR was 0.097, indicating an acceptable result.

### Structural Model and Results Analysis

**FIGURE 2:**
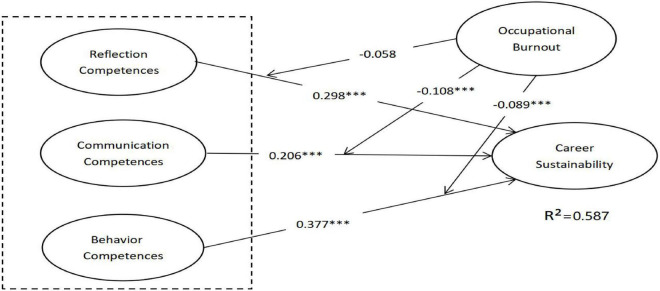
Partial least square (PLS) results of the research model. ^***^t > 1.96.

A bootstrap technique resampling procedure of the partial least square (PLS) was employed to test hypotheses in our study, using resamples of 5,000 (Hair et al., 2017). The result is shown in Figure 2. The overall *R*^2^ value was 0.587, indicating that the research model has a favorable explanatory power as it explained 58.7% of the variance in CS and provided substantial support for H1a–H1c and H2a–H2c.

The empirical results supported hypotheses H1a, H1b, and H1c. Our findings suggested that RCs are positively related to CS (H1a, β = 0.298, *p* < 0.01); CC was positively related to CS (H1b, β = 0.206, *p* < 0.01); and BC was positively related to CS (H1c, β = 0.377, *p* < 0.01). In sum, each dimension of CCs is significantly positively related to CS. H2a–H2c are to explore the moderating effect of OB on the relationship between the individual dimensions of CCs and CS. According to Figure 2, H2b and H2c receive support. The result revealed that the higher the level of OB, the weaker the link between CC and CS (H2b, β = –0.108, *p* < 0.01) and the higher the level of OB, the weaker the link between BC and CS (H2b, β = –0.089, *p* < 0.01). However, the moderating of the mooring effect on the RC is insignificant (H2c, β = –0.032, *p* > 0.05), and the result thus does not support H2a.

In the study, Effect size *f*^2^ was employed to assess the size effect of moderation. Referring to Hair et al. (2017), the effect size is identified in three kinds: small (*f*^2^ = 0.02–0.15), medium (*f*^2^ = 0.15–0.35), and large (*f*^2^ ≥ 0.35). Our finding shows that moderating effect size of OB on relations between CC and CS is small (*f*^2^ = 0.038); the moderating effect size of OB on relations between BC and CS is small (*f*^2^ = 0.023).

## Discussion

The objective of this article was to investigate how OB moderates the linkage between CS and the three sub-dimensions of CCs. The empirical results in study 2 verified the significance of the positive correlations of RC-CS, CC-CS, and BC-CS that were identified in study 1, which support H1a–H1c. Referring to the literature, empirical pieces of evidence posited that CCs function as a personal resource in the Job Demands-Resources (JD-R) model (Xanthopoulou et al., 2007; Akkermans et al., 2015). These results thereby demonstrated that each dimension of CCs positively affects the employees’ sustainable career, in line with previous findings (Blokker et al., 2019; Baruch and Sullivan, 2022). Moreover, the research results yield interesting findings and revealed no significant effect for OB as a moderator for the relation between RC and CS (H2a invalid) whereas the moderating effect of OB is significant on the relation between CC-CS and BC-CS (H2b and H2c). In support of the insignificant moderating effect of OB on the link between RC and CS, Akkermans et al. (2013) demonstrated how employees who are passionate about their career are self-motivated. According to the authors, passionate employees are motivated by the vision they have for their career and they are not easily distracted by challenging work conditions. Having a clear sense of one’s motivation and qualities may enhance one’s career self-concept. Meanwhile, working conditions have proven to directly affect workplace communication and network and employee behavior. Obviously, the communication among workers is a direct reflection of their working conditions, which also affect workers’ behavior toward their career (Chin and Liu, 2015; Quan et al., 2021). In addition, existing literature suggests that OB negatively moderates the relation between CCs and CS (Pillay et al., 2005; Barthauer et al., 2020). This further confirms that the study of the sub-dimension of CCs is of significance (Kuijpers et al., 2006; Parker et al., 2009), while the scant study has investigated the effects of individual dimensions of CCs on CS.

Overall, our study makes three contributions. First, our research enriches the literature on career development by using a mixed-method research design and techniques. By doing so, we can provide a more holistic picture of understanding the role of OB in the CC-CS mechanisms, which partly fill the gap by adopting non-single methodologies in organizational behavior (Venkatesh et al., 2013; Xu et al., 2021). Second, scholars have called for more empirical studies on the effects of individual dimensions of CCs on career-related outcomes (Chin et al., 2019b; Ren et al., 2020). Our study thus responds to this call. Third, we collected data from Chinese employees working in Vietnam MNCs, which enrich the literature by using a unique sample with a cross-cultural setting in a particular GPHC context.

### Limitations and Future Research

In spite of the inspiring findings this study presents, there are some limitations and suggestions for future research. First, the sample of this study only involved Chinese multinational workers in a cross-cultural context, which will affect the relevance of its findings. Future studies should collect data from employees from different countries. Second, this research uncovered the role of OB in affecting the relation between three sub-dimension of CCs and CS without consideration of the social and demographic differences. According to the existing studies, OB is in relation to the social and demographic, such as age, gender, education, and marital status (Kounenou et al., 2010; Hua et al., 2019; Bu et al., 2020). Obviously, the moderating effect of OB will vary according to different social and demographic characteristics (Adam et al., 2018). Hence, future studies could investigate the demographic characteristics as variables.

## Data Availability Statement

The raw data supporting the conclusions of this article will be made available by the authors, without undue reservation.

## Author Contributions

WZ conceived and designed the research, provided guidance throughout the entire research process, and wrote the main part of the manuscript. JB-P collected the data and wrote the methods section. TC wrote the hypotheses development, methodology, and offered modification suggestions. Y-NS and GA participated in an online survey and helped analyzed the data. All authors contributed to the article and approved the submitted version.

## Conflict of Interest

The authors declare that the research was conducted in the absence of any commercial or financial relationships that could be construed as a potential conflict of interest.

## Publisher’s Note

All claims expressed in this article are solely those of the authors and do not necessarily represent those of their affiliated organizations, or those of the publisher, the editors and the reviewers. Any product that may be evaluated in this article, or claim that may be made by its manufacturer, is not guaranteed or endorsed by the publisher.

## Footnotes

1
http://www.vietchina.org/

2
https://www.wjx.cn/
